# Supplementary material for: The causal role of circulating inflammatory markers in osteoporosis: a bidirectional Mendelian randomized study
Source: Front Immunol. 2024 Jul 18;15:1412298. doi: 10.3389/fimmu.2024.1412298 (PMC11291241; doi:10.3389/fimmu.2024.1412298)
Supplement: Supplementary file 1 [file DataSheet_1.zip › supplementary material/CIM-OSTEOPOROSIS /GCST90274844.csv_forest.pdf]

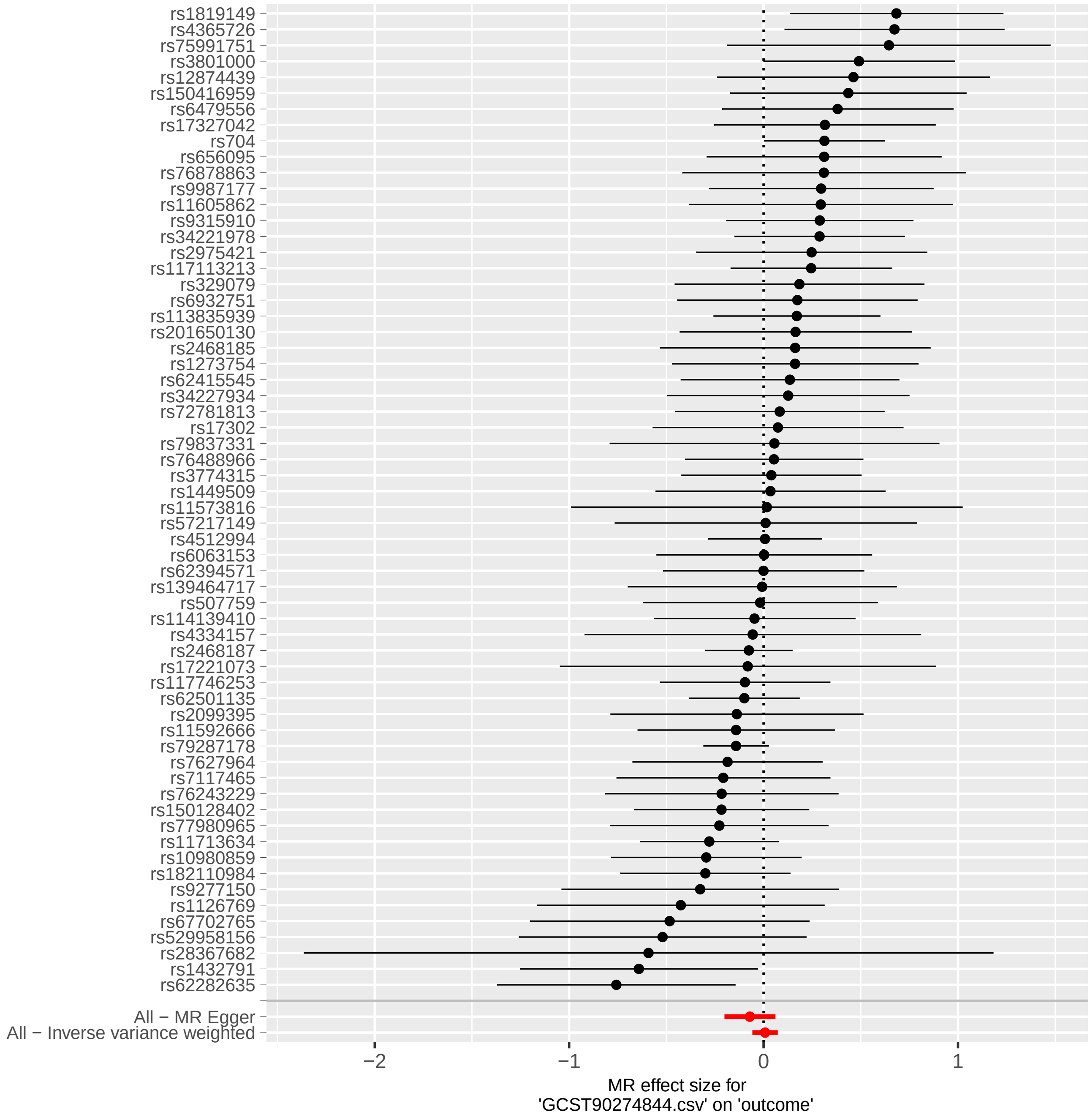
rs1819149
rs4365726
rs75991751
rs3801000
rs12874439
rs150416959
rs6479556
rs17327042
rs704
rs656095
rs76878863
rs9987177
rs11605862
rs9315910
rs34221978
rs2975421
rs117113213
rs329079
rs6932751
rs113835939
rs201650130
rs2468185
rs1273754
rs62415545
rs34227934
rs72781813
rs17302
rs79837331
rs76488966
rs3774315
rs1449509
rs11573816
rs57217149
rs4512994
rs6063153
rs62394571
rs139464717
rs507759
rs114139410
rs4334157
rs2468187
rs17221073
rs117746253
rs62501135
rs2099395
rs11592666
rs79287178
rs7627964
rs7117465
rs76243229
rs150128402
rs77980965
rs11713634
rs10980859
rs182110984
rs9277150
rs1126769
rs67702765
rs529958156
rs28367682
rs1432791
rs62282635
All − MR Egger
All − Inverse variance weighted
−2
−1
0
1
MR effect size for
'GCST90274844.csv' on 'outcome'
